# Knowledge and Confidence in Psychotropic Prescribing, Utilisation of Guidelines and Resources in Managing Perinatal Mental Health in General Practice: An Australian Cross‐Sectional Survey

**DOI:** 10.1111/ajr.70149

**Published:** 2026-01-23

**Authors:** Jacqueline Frayne, Sarah Seddon, Tamara Lebedevs, Talila Milroy, Beverly Teh, Thinh Nguyen

**Affiliations:** ^1^ Medical School, Discipline of General practice The University of Western Australia Crawley Australia; ^2^ Pharmacy Department, King Edward Memorial Hospital Women's and Newborn Health Service Subiaco Australia; ^3^ South Metropolitan Health Services Perth Australia; ^4^ Peel and Rockingham Kwinana Mental Health Services Rockingham Australia; ^5^ Medical School, Discipline of Psychiatry The University of Western Australia Crawley Australia

**Keywords:** clinical practice, guidelines, perinatal mental health

## Abstract

**Objective:**

Management of psychiatric disorders in the perinatal period is a common presentation in primary care. There is a need to understand how general practitioners (GPs) use guidelines and resources and incorporate the knowledge within clinical practice. This study aims to explore Australian GPs' knowledge and confidence in psychotropic prescribing in all stages of pregnancy in accordance with current guideline recommendations.

**Design:**

Cross‐sectional anonymous online survey.

**Setting:**

National GP survey was undertaken.

**Participants:**

132 GPs responded, 84% female, with 41% rural and 59% in metropolitan practice. Main Outcome Measure: exploring knowledge and confidence in psychotropic prescribing, vignettes of a range of clinical scenarios following recommended guidelines, and resource use and screening practices. Exploratory analysis using nonparametric tests occurred between sub‐groups within the data, including zones of practice and GP experience.

**Results:**

Overall, 83% were confident in psychotropic prescribing, a percentage that decreases with more complex and specialised psychotropic medication usage. Rural GPs had higher levels of prescribing knowledge and confidence across scenarios; however, they ranked accessing support from perinatal and general psychiatry lower in treatment choices. Years of experience were not significant. All GPs were aware of clinical prescribing guidelines, with up to 95% having used them. This was less so, with only 37% aware of Australian national perinatal mental health guidelines.

**Discussion:**

GPs have considerable knowledge and confidence and follow best practice recommendations in prescribing common psychotropic medications. The more complex the mental health requirements, the reduced level of confidence and need for greater specialist support. Differences in rural practice locations were observed and could be better served with streamlined support and referral pathways to specialist perinatal mental health advice. Clinical pharmacological practice guidelines were used; however, lack of awareness and usefulness of national guidelines on perinatal mental health could be improved by more specific guidance targeted to a GP population.

## Abstract (highlights)

**What is Already Known on This Subject?:**

- Perinatal mental health contributes to disease burden affecting 1 in 4 pregnancies which are mostly managed in general practice settings
- Most studies focus on perinatal depression rather than more broadly understanding perinatal mental health
- Guidelines exist nationally, but GPs awareness and use of these is unknown.

**What Does This Study Add?:**

- GPs overall confidence and knowledge in managing perinatal mental health are high, reducing with increasing complexity, and is mostly consistent with best practice recommendations
- Rural GPs appear to have more confidence overall and with increased complex scenarios, however the disparity in services across the nation potentially reflected how GPs managed prescribing psychotropic medications, with GPs in metropolitan settings more likely to seek advice from perinatal psychiatrists and treating psychiatrists
- All participants were aware of therapeutic guidelines that they used regularly in their practice, and websites that were related to prescribing information. However, they were less familiar with more overarching Australian perinatal mental health guidelines and found these less useful

## 1 Introduction

Management of psychiatric disorders in the perinatal period is challenging with patients facing complex decisions regarding psychotropic use [1] and health care professionals facing numerous barriers to providing care including concerns around their training and skills [2]. Within Australia, General practitioners (GPs) play a vital role with a 2023 survey on preconception and pregnancy health acknowledging that many women consider ensuring good mental health as highly important during this period [3].

Clinical practice guidelines aim to reduce risk by guiding clinicians with evidence‐based recommendations. These need to be up to date and specifically targeting perinatal mental health to increase effectiveness [4]. Guidelines on mental health care in the perinatal period have been in practice and recently updated in Australia [5] and are originally based on guidelines first published in 2014 from the United Kingdom [6]. A scoping review of the literature on GP experiences around managing perinatal mental health showed that the literature is limited around utilisation of guidelines in clinical practice and that lack of exposure to appropriate resources impacted GPs in their practice [7]. This may be due to many GPs feeling overwhelmed by the number of guidelines and resources for various conditions, and the lack of clear and specific directions for them to be beneficial in individual circumstances [8]. To improve use, there is a need to understand how GPs use current guidelines and resources and incorporate this knowledge, that is their awareness and familiarity with the information, within their clinical practice.

When reporting confidence, a personal evaluation of one's knowledge and application in psychotropic prescribing, a 2020 study comparing perceptions of fetal teratogenicity reported general practitioners have greater confidence than their obstetrician and gynaecologist colleagues, and training and education though adequate in this area could be enhanced [9]. However, there remains limited research on how this confidence differs across various stages of pregnancy and with varying complexity of medication use.

Therefore, this study aims to explore Australian GPs' knowledge and confidence of psychotropic prescribing in all stages of pregnancy. A secondary aim is whether GPs have access to available related resources and treatment guidelines, and whether they perceive these existing sources of information to be useful to their daily practice.

## 2 Method

A convenience sample of 132 GP participants was recruited for a national cross‐sectional survey. Recruitment was via newsletters and emails across Australia, and included the General Practitioner and GP Obstetricians colleges, local networks including hospital shared care databases and across University GP departments to distribute to their networks. Invitation to participate was repeated at two time points. Research ethics was sought through the University of Western Australia. The survey was hosted on Qualtrics (Provo, UT). Data collection took place between January 2022 and June 2023. Participation was anonymous. Participants were not incentivised to participate in the study. However, at the end of the survey they were offered to de‐identify themselves to enter a draw for one of five $100 Coles gift cards.

### 2.1 Materials

A survey was purpose designed to assess GPs' understanding, confidence, and prescribing practice in the perinatal period. The survey contained demographic data, including age, gender, location of practice, defined as metropolitan zone, rural zone, remote zone, or non‐practicing, years of experience in general practice, defined into early (1–10 years), mid (11–20 years), and late (20 plus years) career [10] and level of training or qualifications, simple self‐reported confidence levels, short vignettes to assess treatment preferences based on current guideline recommendations, and free text boxes for participants to add relevant information. The survey underwent multiple iterations and was reviewed and piloted independently by multiple healthcare professionals (psychiatrists, pharmacists, and GPs) for clarity and accessibility. The STROBE guidelines were adhered to for this cross‐sectional survey.

### 2.2 Survey Development

Confidence rates are collected routinely in research, and in this survey two measures were used, a 5‐point Likert scale and a 100‐point scale. These tools were selected for convenience and regardless of choice of point scale are determined as being comparable [11]. Participants were asked to rate their confidence in consulting, advising, and prescribing for psychiatric disorders in general using a 5‐point scale (1 = *very uncomfortable*, 5 = *very comfortable*). When initiating and switching medication, participants were asked to rate their levels of confidence in initiating SSRIs, SNRIs, anxiolytics, antipsychotics, and anticonvulsants at any stage during the perinatal period, during preconception, first trimester, second/third trimester, and breastfeeding using a scale of zero (*very uncomfortable*) to 100 (*very comfortable*).

Vignettes are an acceptable methodology to assess clinicians' knowledge and skills in decisional making [12] with each vignette assessing differing psychotropic medication factors. Construct validity and approximation in key aspects of the scenario to real world situations were enhanced through development of these with GPs and psychiatry input. Treatment vignettes were created from four theoretical but realistic scenarios that GPs might encounter in practice. Participants were given a list of treatment options for each scenario and asked to rank each item from *first‐line treatment* to *last treatment option*. Treatment options were based on best practice guidelines for the specific disorders. A fifth scenario was provided; however, in this instance, participants were asked to rate their agreement with four statements related to the patient's treatment. Agreement was rated on a scale of zero (*Disagree*) to 100 (*Agree*).

Participants were asked to rate six considerations for prescribing psychotropic medication from 1 (*most influential*) to 6 (*least influential*). Those considerations were: known safety profile, experiences in prescribing this medication, patient's prior responsiveness to the medication, cost of medication/coverage under PBS, interactions with other medications, and side effect profile of the medication. Participants were given a list of common barriers to providing non‐pharmacological support, e.g., wait periods, cost of services, and limited consultation time. Participants were also provided a free‐text box to indicate other barriers they had encountered which were not listed.

Understanding around knowledge of resources allowed for Australian and national resources covering perinatal and therapeutic treatments. Participants were asked to indicate whether they were unaware or aware of the resource, had used the resource, found the resource useful, and how frequently they viewed the information in the resources. A free‐text box was provided to include any resources not listed.

Participants were asked to rate five risk factors from zero (*not at all important*) to 100 (*extremely important*) associated with screening for depression. Further, they were asked to indicate if they commonly used the Edinburgh Postnatal Depression Scale (EPDS), Kessler Psychological Distress Scale (K10), Depression Anxiety and Stress Scale (DASS‐21), or Antenatal Risk Questionnaire (ANQR) to screen. A free‐text box was provided to indicate a screening tool they use, which was not listed. As a follow‐up question, participants were asked to indicate how often they use screening tools for assessing postnatal depression: > 80%, 60%–80%, 40%–60%, 20%–40%, or < 20%.

### 2.3 Data Analysis

Missing value analysis was conducted. Data did not appear to be influenced by our groups of interest: years of practice and zone of practice. As we were interested in exploratory analysis and differences between sub‐groups within the data and outcomes, data imputation was not used for missing responses. Instead, missing data was excluded from analyses on a case‐wise basis. Descriptives were calculated using SPSS (Version 29) to gain an overall understanding of the sample and their perceptions and experiences when prescribing in the perinatal period. Second, we explored two groups of interest: years of practice and location of practice. The sample violated tests of normality. As such, non‐parametric tests were used to compare the responses between groups of interest. Mann–Whitney *U* tests were used when comparing the dichotomised variable zone of practice with ordinal variables. Kruskal‐Wallace *H* test was used to compare differences in responses based on years of practice and ordinal variables, with Bonferroni corrections for multiple comparisons. Due to an inadequate sample size for the chi‐square test of homogeneity, Fisher's exact tests were used to compare differences between the above‐mentioned groups of interest and nominal variables.

## 3 Results

A total of 163 responses were recorded. Of these responses, 15 were removed as only the consent statement had been completed and no other questions. A further 16 were removed as respondents had only completed the first two demographic questions before exiting the survey. A sample of 132 general practitioners (GPs) from across Australia completed the survey. Participants were predominantly female (84.1%), and most reported working within the metropolitan zone of practice (59.1%). Most participants reported completing further studies in women's health, family health, or general mental health (87.9%). Lastly, most participants reported that 40% to 60% of their patients included women of reproductive age. A detailed breakdown of the sample characteristics can be found in Table 1.

**TABLE 1:** Demographics, zone and experience of practice of survey sample (*N* = 132).

	Frequency *n* (%)
Age	
< 30	2 (1.5)
31–40	39 (29.5)
41–50	47 (35.6)
51–60	27 (20.5)
> 61	17 (12.9)
Gender	
Male	21 (15.9)
Female	111 (84.1)
Location of practice	
Metropolitan Zone	78 (59.1)
Rural Zone	48 (33.3)
Remote Zone	8 (6.1)
Non‐practicing	1 (0.8)
Years in general practice	
1–10	51 (38.7)
11–20	34 (25.8)
> 20	47 (35.6)
Capacity of work in general practice	
Full‐time	46 (34.8)
Part‐time	81 (61.4)
Locum	4 (3)
Non‐practicing	1 (1)
Current level of training	
Non‐Vocational GP	1 (0.8)
GP Registrar	3 (2.3)
Fellow	103 (78)
Certificate of Women's Health	16 (12.1)
Diploma/Advanced Diploma in Obstetrics	84 (63.6)
Level 1 or Level 2 in Mental Health Skill Training	70 (53)
Diploma/Master of Psychiatry	1 (0.8)
Other	
Diploma of Child Health	3 (2.4)
Focused Psychological Skill Training (RACGP)	1 (0.8)
Vocational Graduate Diploma: Women's Health	1 (0.8)
IBCLC	1 (0.8)

*Note:* The percentage is larger than 100 due to multiple responses per participant.

Abbreviation: IBCLC, International Board of Lactation Consultant Examiners.

### 3.1 Confidence

#### 3.1.1 Psychiatric Disorders in General

Most participants rated themselves as *somewhat comfortable* (54.5%) or *very comfortable* (28.8%) when asked about their confidence in consulting, advising, or prescribing for psychiatric disorders. There were no significant differences between zone of practice (between metropolitan or rural regional and remote), or years of practice and levels of confidence in consulting, advising, or prescribing for psychiatric disorders. Table 2 represents confidence levels with prescribing and differences between metropolitan or rural regional and remote practice and years of practice.

**TABLE 2:** Median Confidence in initiating and switching psychotropic medications.

	Zone of practice		*p*	Years of practice experience			*p*
	Metropolitan	Rural, regional, remote		1–10	11–20	> 20	
Initiating specific medications							
SSRI	96	100	0.247	88.5	100	99.5	0.265
SNRI	52.5	75.5	0.007*	52	79	52.5	0.005*
Anxiolytics	31	50	0.281	26	77	48	0.303
Antipsychotic	25	49.5	0.004*	27.5	42	25	0.154
Anticonvulsant	4.5	23	0.027*	3.5	22	5.5	0.590
Initiating during specific stages of the perinatal period							
Preconception and planning	77	76	0.931	76	83	76.5	0.590
First trimester	69	69.5	0.726	65	76	60	0.062
Second and third trimester	72	78	0.397	69	78	75.5	0.538
Breastfeeding	80	88.5	0.367	81	87	78.5	0.505
Switching specific medications							
SSRIs	76	86.5	0.061	76.5	87	82	0.515
SNRIs	62	78	0.058	59	78	78	0.163
Anxiolytics	52	57	0.439	51	78	51	0.034*
Antipsychotics	23.5	46	0.010*	24	25	26	0.541
Anticonvulsants	8.5	30.5	0.006*	6.5	23	22.5	0.292

*Note:* Median level of confidence measured from 0 = very uncomfortable to 100 = very comfortable.

Abbreviations: SNRI, selective noradrenaline reuptake inhibitor; SSRI, selective serotonin reuptake inhibitor.

* Significant at *α* = 0.05, Bonferroni adjusted significance values are reported.

#### 3.1.2 Initiating Specific Medication

Higher confidence levels in initiating medications were reported for SSRIs (*Mdn* = 99) and SNRIs (*Mdn* = 66.5). Initiating anxiolytics (*Mdn* = 42) and antipsychotics (*Mdn* = 27.5) were rated between *somewhat uncomfortable* and *neutral*. Anticonvulsants had the lowest reported confidence (*Mdn* = 8), indicating that participants were less than *somewhat uncomfortable* when initiating this class of psychotropic medication.

When comparing the zone of practice and levels of confidence in initiating psychotropic medication, participants who reported working in rural, regional, or remote zones reported significantly higher levels of confidence in prescribing SNRIs (*Mean Rank =* 74.25, *p* = 0.007, *r* = 0.24), antipsychotics (*Mean Rank =* 68.45, *p* = 0.004, *r* = 0.27), and anticonvulsants (*Mean rank =* 60.86, *p* = 0.027, *r* = 0.22) compared to those who worked in metropolitan areas (*SNRI Mean Rank* = 56.43, *Antipsychotic Mean Rank* = 50.10, *Anticonvulsant Mean Rank =* 47.55). However, the effect size of these differences is small.

When comparing years of practice and confidence levels, only initiating SNRIs appeared to differ between groups significantly. Specifically, participants who reported 11–20 years of practice had significantly higher (*Mean Rank* = 82.41) confidence levels than those who reported more than 20 years of experience (*Mean Rank* = 57.34, *p* = 0.009, *r* = 0.33). Further, those who reported 11–20 years of experience reported significantly higher confidence prescribing SNRIs than those who reported 1–10 years of experience (*Mean Rank* = 59.16, *p* = 0.016, *r* = 0.302). There was no significant difference in confidence prescribing SNRIs between those who reported greater than 20 years of practice and those who reported 1–10 years of practice. Both significant differences yielded medium effect sizes.

#### 3.1.3 Initiating Medications in Specific Perinatal Periods

In general, the highest levels of confidence in initiating medication were reported in the preconception stage (*Mdn* = 76.5) and in breastfeeding (*Mdn* = 82). Initiating medications in the first trimester (*Mdn* = 69) and second and third trimesters (*Mdn* = 73) were rated between *neutral* and *somewhat comfortable*.

When comparing zones of practice and levels of confidence when initiating in specific perinatal periods, there were no significant differences between metropolitan zones of practice and rural, regional, or remote zones of practice. When comparing years of practice and confidence initiating medications within specific perinatal periods, there were no significant differences between groups.

#### 3.1.4 Switching Specific Medications

As with initiating specific medications, the highest confidence levels when switching medications were reported for SSRIs (*Mdn* = 78), followed by SNRIs (*Mdn* = 70). Switching anxiolytics (*Mdn* = 54.5) was rated as roughly *neutral*. Antipsychotics (*Mdn* = 25) and anticonvulsants had the lowest reported confidence (*Mdn* = 22) and were rated closer to *somewhat uncomfortable*.

When comparing the zone of practice and levels of confidence in switching psychotropic medication, participants who reported working in rural, regional, or remote zones reported significantly higher levels of confidence in switching antipsychotics (*Mean Rank* = 66.28, *p* = 0.010, *r* = 0.25) and anticonvulsants (*Mean Rank* = 62.75, *p* = 0.006, *r* = 0.26) compared to those who worked in metropolitan areas (*Antipsychotic Mean Rank* = 50.17, *Anticonvulsant Mean Rank* = 46.10). Though significantly different, the magnitude of the effect size was small.

When comparing years of practice and confidence with switching medications, participants who reported 11–20 years of practice reported significantly higher confidence (*Mean Rank* = 76.33) compared to those who reported 1–10 years of experience (*Mean Rank* = 56.40, *p = 0.043, r = 0*.27). However, the effect size was small.

### 3.2 Treatment

Vignette responses are presented in Table 3 being ranked in the order of treatment options aligned with best practice guidelines and comparing zones of practice—metropolitan and rural & remote. For *Vignette 1*, describing mild to moderate depression in the first trimester, the ordering of choices aligned with best practice guidelines. *Vignette 2* described a category D medication in the first trimester with stable mental health. There were differences between where the groups ranked calling the treating psychiatrist (Metro = 2, Rural = 3, *p* = 0.010), calling a perinatal psychiatrist (Metro = 4, Rural = 5, *p* = 0.003), and where they would rank tapering, stopping, and switching the medication (Metro = 5, Rural = 6, *p* = 0.001). *Vignette 3* described a woman with bipolar one on lithium in the first trimester. There was a significant difference in where these groups ranked their choice of tapering, stopping, and switching medications (*p* = 0.023). GPs in the rural, regional, and remote zones commonly ranked this as their third line of treatment, whereas GPs in the metropolitan area commonly ranked this as their fifth line of treatment. The groups also significantly differed in where they ranked calling a perinatal psychiatrist for advice (*p* < 0.001). *Vignette 4* described the use of dexamfetamine in the third trimester. Results indicated significant differences in ranking of treatment between zone of practice with metropolitan workers ranked calling the treating psychiatrist higher in choice than rural, regional, and remote GPs (*p* = 0.017). There was also a significant difference in the distribution of rank choices when it came to tapering, stopping, and switching medications (*p* = 0.008). Finally, *Vignette 5* ranked statements in regard to venlafaxine exposure in the third trimester. There were no significant differences in agreement when comparing zone of practice.

**TABLE 3:** Treatment vignettes ranking treatment options comparing metropolitan and rural zones of practice.

Vignette	Treatment options ranked in order of importance and aligned with clinical guidelines (1 highest–7 lowest)	Percentage of GPs that ranked the treatment option in this position	
		Metropolitan	Rural/remote
1: A woman is seen at your practice who is 10 weeks pregnant. During your consultation, you diagnose the woman with mild/moderate depression.	Provide psychoeducation and review Refer the woman for psychological support Trial with an antidepressant Refer to a psychiatrist for opinion management. Leave to an O&G for management.	70 68 80 69 80	86 81 83 77 89
2: A woman with a 10‐year history of stable OCD is currently 6 weeks pregnant. Her medications include 20 mg of paroxetine daily, which your software informs you is a category D medication. What would your management be?	Counsel on risks/benefits of treatment. Discuss with her treating psychiatrist Discuss with a perinatal psychiatrist Reduce the dosage of medication Taper and stop the medication and consider an alternative SSRI Discuss with a tertiary pregnancy pharmacy information service Stop her current medication as it is a teratogen	46 32* 29* 13 24* 19 68	56 23* 13* 17 19* 17 58
3: A woman with established Bipolar I Disorder under psychiatric care presents for routine blood tests. She is 9 weeks pregnant and currently taking lithium carbonate.	Counsel on risks/benefits of treatment. Discuss with her treating psychiatrist Discuss with a perinatal psychiatrist Discuss with a tertiary pregnancy pharmacy information service Reduce the dosage of medication Taper and stop the medication and consider an alternative Stop her current medication as it is a teratogen	36* 39 37* 15 36 31 50	50* 40 25* 3 19 29 54
4: A woman with ADHD attends your practice for a routine consult for a medical certificate. During the consult, the patient informs you that she is 30 weeks pregnant and also taking 60 mg of dexamfetamine per day.	Counsel on risks/benefits of treatment Discuss with her treating psychiatrist Discuss with a perinatal psychiatrist Discuss with a tertiary pregnancy pharmacy information service Reduce the dosage of medication Taper and stop the medication and consider an alternative Stop her current medication as it is a teratogen	46 38* 33 21 51 62* 68	56 31* 29 23 44 46* 71

Vignette	Treatment options ranked in order of agreement	Rank agreement	
5: A woman attending your practice is on venlafaxine 300 mg XR. She is 36 weeks pregnant and asks you what she should do about her medication. There are many ways that this might be managed.	Venlafaxine is safe for breastfeeding She should give birth at a centre with access to paediatric care due to the high risk of neonatal abstinence syndrome She should remain on her current dose without change to prevent postnatal depression Reducing the dose of Venlafaxine has consistently shown benefits in reducing neonatal abstinence syndrome.	*Mdn =* 74 *Mdn =* 65 *Mdn =* 56 *Mdn =* 54	*Mdn =* 76 *Mdn = 54* *Mdn = 65* *Mdn =* 54

Abbreviations: ADHD, attention deficit hyperactivity disorder; OCD, obsessive compulsive disorder.

* *p* < 0.05 statistically significant.

### 3.3 Considerations of Treatment

The known safety profile of the medication was ranked by 69.5% of the sample as the most influential factor when prescribing psychotropic medications. GPs' experience with prescribing the medication was ranked by 35.94% as the second most influential factor. The patient's prior experience was ranked as the second (35.16%) and third (36.72%) most influential factor. Forty‐two per cent ranked the side effect profile as the fourth most influential factor. The possible interactions with other medications were fifth‐ranked, by 44.53% of the sample. The cost of the medication was ranked with 67.72% of GPs as the least influential factor when prescribing psychotropic medications. The ranking of influential factors did not significantly differ when comparing zones of practice or years of experience.

### 3.4 Knowledge of Resources

Participants were able to choose multiple options for these questions. For clarity, participants who had indicated, in any combination, that they found a resource useful were grouped with the general *found useful* response. Participants who indicated that they were aware of a resource and had used it were grouped with the general have‐used response. This aims to distinguish between being aware of a resource, using that resource, and having found that resource useful.

#### 3.4.1 Resources

The knowledge and use of resources are shown in Table 4. The most known Australian resource amongst GPs who answered this question was the Therapeutic Guidelines. Of the GPs who had reported using this guideline, 92% reported it was useful. Only 36.6% of GPs reported being aware of the COPE guidelines [5]. Of the international guidelines listed, most GPs reported being aware of the NICE [6] guidelines (76.3%). However, of the 66 GPs who had reported using them, only 39 reported finding them useful. Overall, with the use of guidelines, most respondents, 67.4%, indicated that they review the information in these guidelines as needed/when they have a patient. The next largest percentage, 15.5%, reviewed them once a week. Further resources that were identified as useful by GP participants were: local hospital guidelines, hospital pharmacy phoneline services, embedded GP Software information, clinical education resources, and breastfeeding resources.

**TABLE 4:** Knowledge of resources.

	Unaware	Aware	Used	Found useful
Australian resources				
COPE (*N* = 131)	63.4% (83)	36.6% (48)	39.6% (19)	63.2% (12)
Therapeutic Guidelines (*N =* 131)	0% (0)	100% (131)	95.4% (125)	92% (115)
NPS Medicines (*N* = 130)	0.77% (1)	99.2% (129)	91.5% (118)	76.3% (76.3)
Australian Medicines Handbook (*N* = 131)	1.5% (2)	98.5% (129)	73.6% (95)	63.16% (60)
International resources				
MothertoBaby.Org (*N* = 131)	67.2% (88)	32.8% (43)	39.5% (17)	58.8% (10)
WomensMentalHealth.Org (*N* = 129)	84.5% (109)	15.5% (20)	30% (6)	50% (3)
NICE Guidelines (*N* = 131)	23.6% (31)	76.3% (100)	66% (66)	59.1% (39)
MotherSafe (*N* = 128)	46.9% (60)	53.1% (68)	58.8% (40)	75% (30)
Bumps/UKTIS (*N* = 128)	89.1% (114)	10.9% (14)	7.1% (1)	0% (0)
LactMed (*N* = 130)	40% (52)	60% (78)	57.7% (45)	53.3% (24)

*Note:* Table percentages do not equal 100% due to the iterative nature of the question's answering style.

#### 3.4.2 Screening for Perinatal Mental Health

In general, participants rated all considerations as risk factors for perinatal mental health above *moderately important* on a 0 to 100 scale of importance. The consideration which received the highest importance was *previous history of perinatal depression*, which had a median rating of 100. Next was *previous history of trauma* with a median rating of 96. Next in importance was *relationship with partner* (Mdn = 80) and then *pregnancy and birth complications* (Mdn = 77). The aspect that was reported least important was *family history of depression*, which was rated slightly above *moderately important* at a median of 55. Interestingly, when comparing years of practice, those who had worked 5–10 years rated *pregnancy and birth complications* (Mdn = 79.31) higher than those who had worked less than 5 years (Mdn = 46.74, *p* = 0.026). Further, those who had worked 11–15 years rated *previous history of trauma* (Mdn = 85.21) higher than those who reported 16–20 years of experience (Mdn = 40.79, *p* = 0.006).

The Edinburgh Perinatal Depression Scale (EPDS) was the most used brief screening tool (*N* = 114). This was followed closely by the Depression, Stress, and Anxiety Scale –21 item (DASS‐21) (*N* = 60) and the Kessler Psychological Distress Scale (K10) (*N* = 59). Only three participants reported using the Antenatal Risk Questionnaire (ANRQ). Of the measures used but not listed, five indicated using the Kimberly Mums Mood Scale, one person indicated using the Wakefield Depression Scale, and three indicated they would use supportive care, mental health assessment, or clinical assessment as a tool.

Almost half (47.7%) indicated that they use screening tools to assess for postnatal depression more than 80% of the time. Approximately 10.6% of the respondents indicated they use them less than 20% of the time when screening for postnatal depression.

### 3.5 Barriers to Providing Non‐Psychotropic Support

The highest reported non‐pharmacological barrier to providing support was *wait period of services* with 90.2% of the sample endorsing this option. This was followed by *cost of service* (87.25%) and the *limited GP consultation time* (62.4%). Interestingly, *lack of knowledge of local services* (24.1%) and *limited GP training in this area* (15.8%) received the lowest endorsement. Of other aspects not listed, eight participants indicated a lack of access to services in part due to distance and rural location. Others indicated that turn‐over of clinical psychologists or psychologists lacking perinatal mental health experience was an issue. Finally, lack of indigenous services and the poor patient experience of calling services to get an appointment were also indicated as non‐pharmacological barriers.

## 4 Discussion

Overall, our study has shown that general practitioners reported high levels of confidence in the management of perinatal mental health. A significant finding in our study was the differences in rural and metropolitan practices, with GPs working in rural settings having increased confidence in both initiating and switching medications. Confidence in prescribing overall reduced with increasing complexity around the classes of prescribed medications. GPs had most confidence in prescribing in the postnatal period and this may be due to their awareness of good safety data with breastfeeding [13]. Alternatively, consistent with another Australian survey of GPs and obstetricians by Williams et al. in 2021 [9], concerns regarding teratogenic effects remain, with confidence lowest with prescribing in the first trimester of pregnancy in our sample. This is seen in vignettes 2 and 3 where category D drugs are prescribed. This highlights some of the challenges with the current categorisation of drugs in pregnancy, and further advocates for awareness of a weighing up of individual risk and benefits approach [14] being first line. Further, survey participants ranked safety around medication as the key priority when counselling women.

When considering how and what to prescribe for women in the perinatal period, GPs' knowledge around medication seemed overall consistent with current recommendations, with the safety profile of medications and patients' past experiences ranking high. This was also shown regarding tapering, stopping, or switching medications. Participants ranked ceasing medication, even lithium, a potential teratogenic agent, as low without first fully exploring other treatment avenues or seeking specialist support. This is important as lithium prescribing in pregnancy is seen as an effective treatment for the prevention of relapse in bipolar disorder [15].

The disparity in services across the nation potentially reflected how GPs managed prescribing psychotropic medications, with GPs in metropolitan settings more likely to seek advice from perinatal psychiatrists and treating psychiatrists in general with the more complex vignettes. We remain uncertain why higher confidence in prescribing in the rural setting was seen, but this may be due to it being a necessity with limited specialist access. Barriers to non‐pharmacological care were primarily due to access to services with wait times and costs for patients, including mention of rural and distance factors impacting this. These highlight a need reflected in the Australian and New Zealand College of Psychiatrists position statement where recommendations call for increased access to antenatal consultant liaison for women with more severe mental health needs regardless of geographical location [16] and a call to improve cost and access to psychological support for perinatal care across the country.

The use of guidelines and resources by GPs was variable. All participants were aware of therapeutic guidelines that they used regularly in their practice, and websites that were related to prescribing information. However, they were less familiar, with just over a third of participants aware of more overarching Australian [5] and 76% aware of the NICE UK guidelines [6] for perinatal mental health. Of those who used these guidelines, around 60% found they were useful. This may be due to the guidelines being not specific or easy to access or use in GP settings; however, this finding was not explored further in this survey. Further exploration of this would be helpful in ongoing research.

Our findings of a non‐significant difference between years of practice and levels of confidence in prescribing may reflect the impact of training in this sample. Most participants had some level of additional training in either women and/or mental health. However, there were some subtle differences in the level of experience in this study that are possible a result of training influences over time. For example, known risk factors for perinatal depression aid identification, and include a past history of mental illness, socioeconomic status, domestic violence and substance use [17]. When identifying women at risk of perinatal mental health issues, GPs' clinical experience, with those in the middle years of their career, seemed more cognizant of the impact of pregnancy and birth complications and a history of trauma. Birth‐related trauma is seen at higher levels in those experiencing perinatal mental health events in the postpartum period [18], and raised awareness around the importance of adverse childhood experiences and the impact on the health and wellbeing of patients seen in general practices [19] has occurred in recent years, along with the use of the antenatal risk questionnaire (ANRQ) in some parts of Australia as a self‐reported measure of psychosocial risk prediction for postnatal depression [20]. Even though only a few of our participants used the ANRQ it is possible they may have been exposed to this during hospital maternity training.

The EPDS was the most frequently used tool by GPs, but not the only one for screening purposes. Almost half of survey participants used these tools regularly in their practice. Current consensus guidelines recommend that all women should preferably complete this twice, once antenatally and ideally in the postpartum period [5]. While our results are promising in showing use of screening in practice, a greater understanding around the use of these tools by GPs in the primary care setting is needed.

Strengths and limitations of this study relate to the demographic and sample size. While not representative of all GPs due to the elevated number of female participants and those with extra skills training in women's health or obstetric care, it captured a population of GPs managing women over their reproductive life course and providing maternity care. A strength of this study was seen in that the numbers of participants working across rural, regional and remote centres were sufficient to compare differences in GPs prescribing practices. Further vignettes, while capturing guideline recommendations, failed to provide further context to understanding their reasons for ranking. While some of the differences were statistically significant, the clinical relevance of these differences was not addressed here and would warrant further investigation through more focused questionnaires or qualitative interviews. Additionally, a priori power analyses were not conducted prior to data collection for this research. As such, interpretation of non‐significant findings must be done with caution.

## 5 Conclusion

Overall GPs have considerable knowledge and confidence in prescribing common psychotropic medications. The more complex the mental health requirements, the reduced level of confidence and need for greater specialist support. Differences occurred in GPs practicing in the rural setting compared to metropolitan areas and calls for increased access with streamlined support and referral pathways to specialist perinatal mental health advice across the nation. Clinical pharmacological practice guidelines were known and used by GPs; however, lack of awareness and the usefulness of national guidelines on perinatal mental health could be improved by delivering tailored national guidance targeted to a GP population specifically. Incorporation of guidelines using digital tools, decision trees or integrated software could improve access and use.

## Author Contributions

**Jacqueline Frayne:** conceptualization (lead), writing – original draft (lead), methodology, investigation, formal analysis, writing – review and editing. **Sarah Seddon:** software (lead), investigation, formal analysis (lead), writing – review and editing. **Tamara Lebedevs:** conceptualization (supporting), methodology, writing – review and editing. **Talila Milroy:** investigation, writing – review and editing. **Beverly Teh:** investigation, writing – review and editing. **Thinh Nguyen:** conceptualization (supporting), methodology, writing – review and editing.

## Funding

This work was supported by the Royal Australian College of General Practitioners, TGL2021‐02.

## Ethics Statement

Research ethics was sought through the University of Western Australia (2021/ET000989).

## Conflicts of Interest

The authors declare no conflicts of interest.

## Data Availability

The data that support the findings of this study are available from the corresponding author upon reasonable request.
